# Supplementary material for: Tunable and switchable magnetic dipole patterns in nanostructured superconductors
Source: Nat Commun. 2018 Jul 3;9:2576. doi: 10.1038/s41467-018-05045-3 (PMC6030140; doi:10.1038/s41467-018-05045-3)
Supplement: Supplementary file 1 — Supplementary Information [file 41467_2018_5045_MOESM1_ESM.pdf]

SUPPLEMENTARY INFORMATION

Tunable and switchable magnetic dipole patterns in nanostructured superconductors

Ge et al.

Junyi_Ge@t.shu.edu.cn
victor.Moshchalkov@kuleuven.be

Supplementary Figures

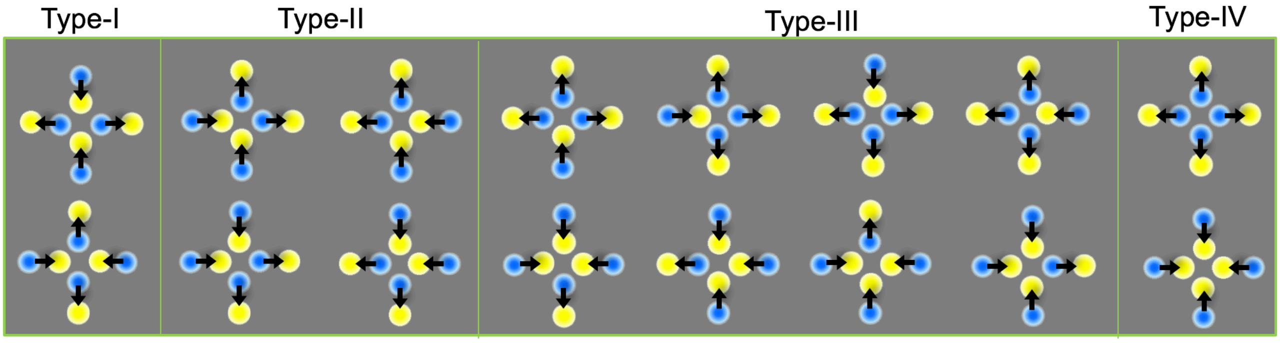

Supplementary Figure 1: Various vertex configurations in a square spin ice. The vertex configurations are classified into four types in order of increasing energy. The yellow/blue circles indicate the positive/negative magnetic charges corresponding to each macrospin (arrows).

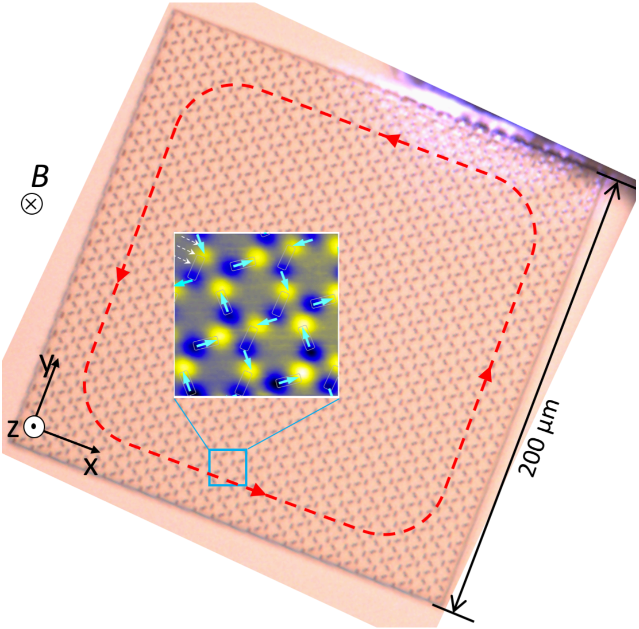

Supplementary Figure 2: The optical image of the measured sample. The scanned area is indicated by the blue square close to the center of one sample edge. The red dashed line schematically shows the flowing Meissner current, which is induced by applying an external magnetic field. Since the Meissner current flows along the border of the sample and changes direction at opposite edges, the observed magnetic dipoles also change their polarities accordingly. In our manuscript, all the SHPM images were measured at the same position indicated by the blue square. The inset shows the same image as Fig. 1e in the manuscript.

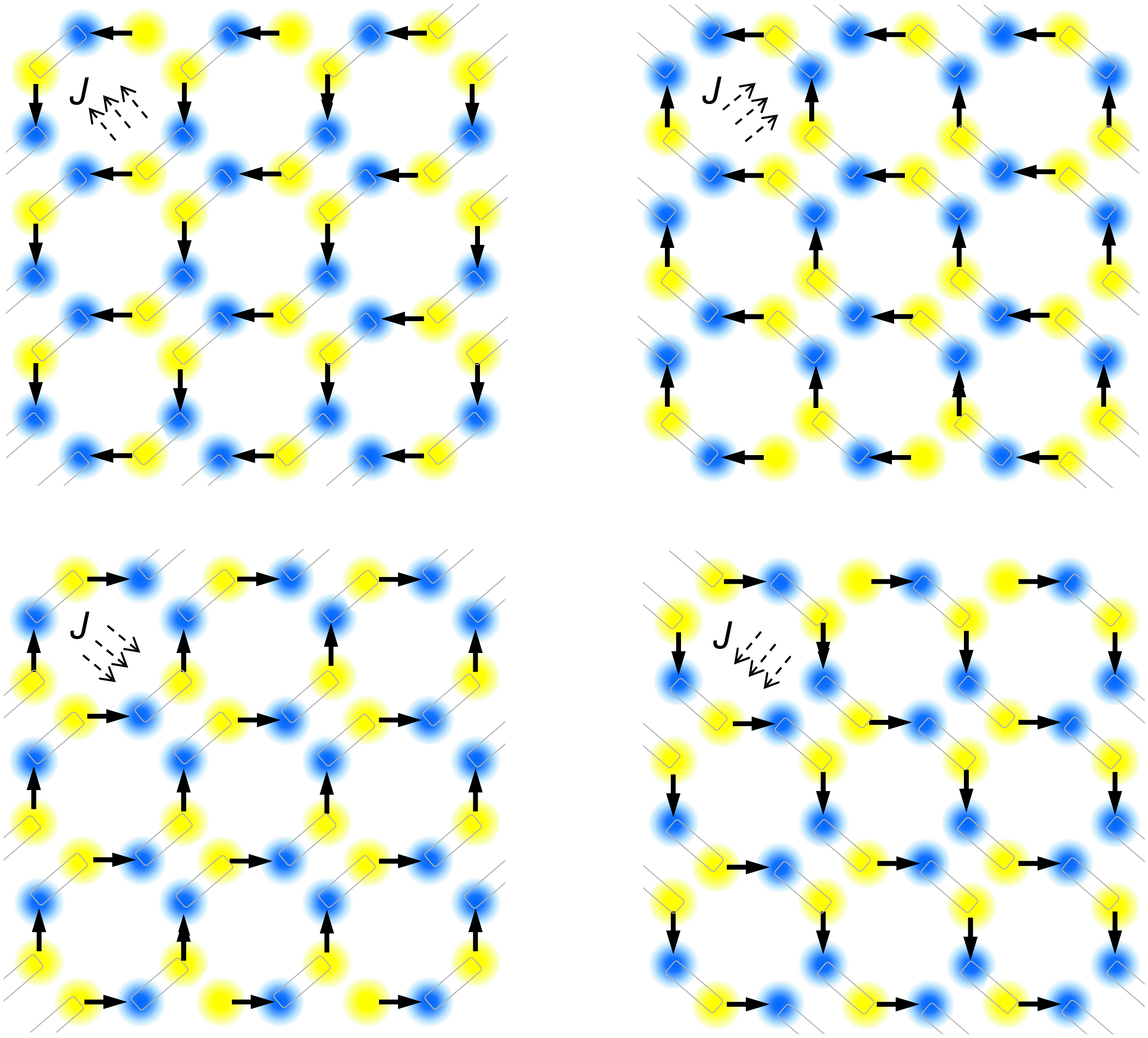

Supplementary Figure 3: Realization of Type-II ice-like magnetic states with magnetic dipole pattern. The rectangles indicate antidots formed in a superconductor. The solid arrows show the spin distribution for type-II vertex configurations of a square spin ice. The dashed arrows indicate the direction of flowing supercurrent. The dimension of the rectangles is 2.4 μm × 0.8 μm.

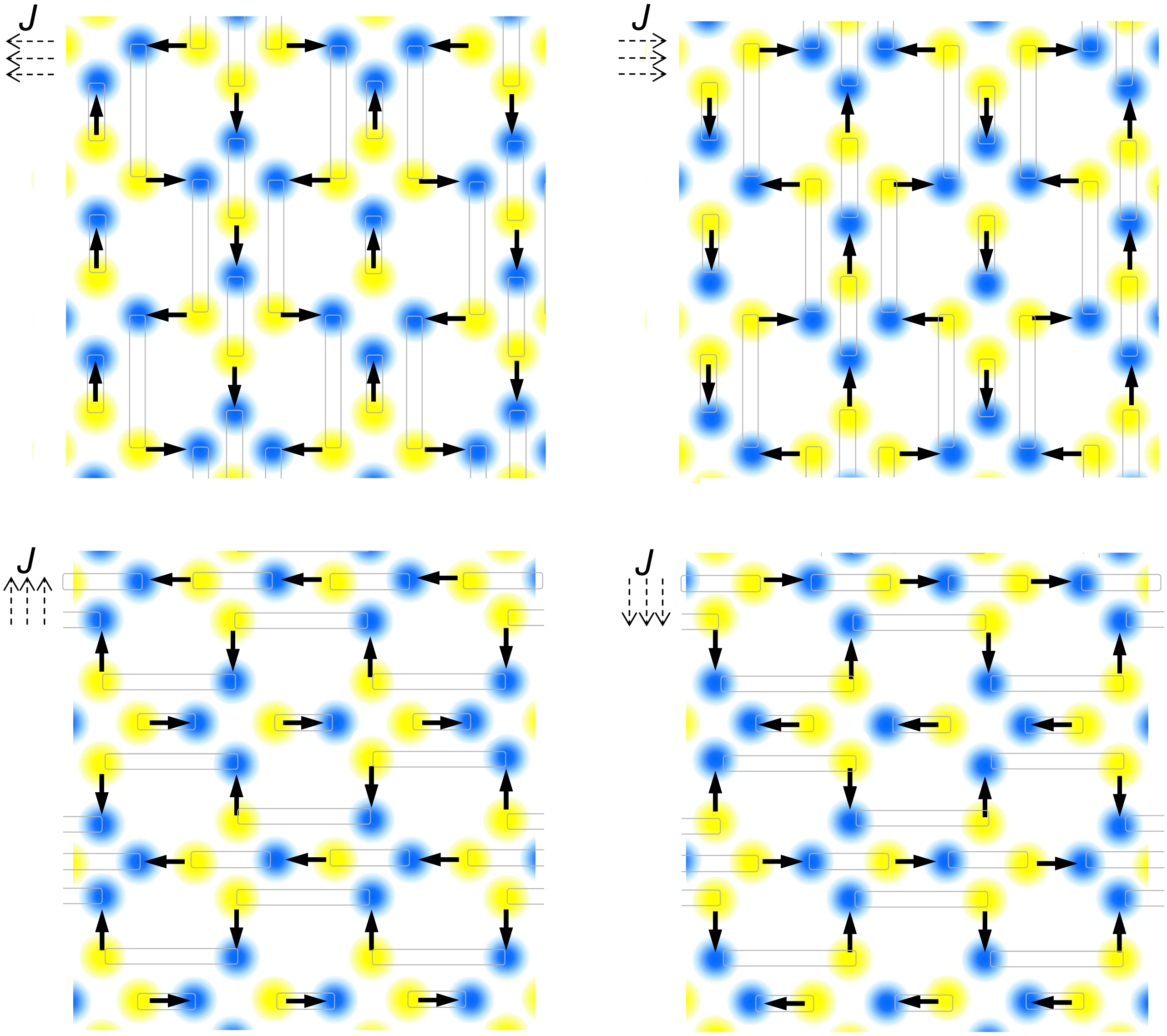

Supplementary Figure 4: Realization of Type-III ice-like magnetic states with magnetic dipole pattern.. The rectangles indicate antidots in the superconductor. The solid arrows show the spin distribution for type-III vertex configurations of a square spin ice. The dashed arrows indicate the direction of flowing supercurrent. In the upper two images, the long (short) rectangles have the dimensions of 3.4 µm × 0.8 µm (5.8 µm × 0.8 µm).. In the lower two images, the long (short) rectangles have the dimensions of 5.8 µm × 0.8 µm (2.4 µm × 0.8 µm).

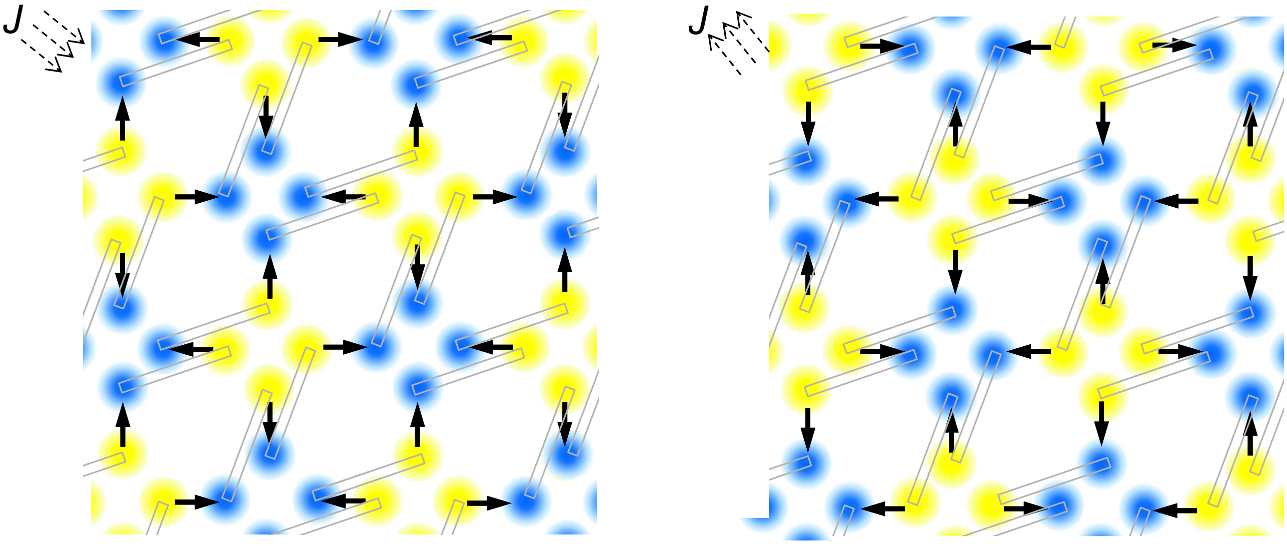

Supplementary Figure 5: Realization of Type-IV ice-like magnetic states with magnetic dipole pattern.. The rectangles indicate antidots in the superconductor. The solid arrows show the spin distribution for type-IV vertex configurations of a square spin ice. The dashed arrows indicate the direction of flowing supercurrent. The rectangles have the dimensions of 4.4 μm×0.8 μm.

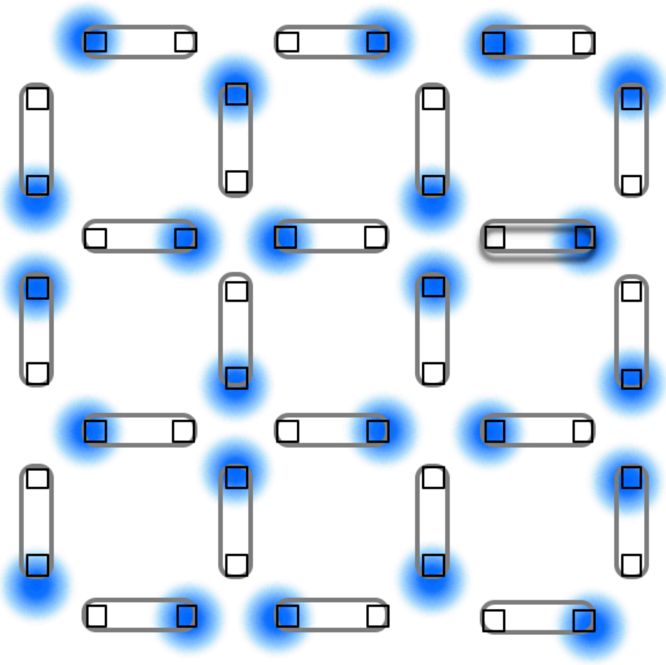

Supplementary Figure 6: Schematic image of the vortex ice ground state. For vortex ice, four pairs of antidots (squares) meet at one vertex. At half matching field, there is only one antidot occupied by one vortex (blue area). At each vertex, the ground state distribution requires the vortices to form two-close/two far way configuration.

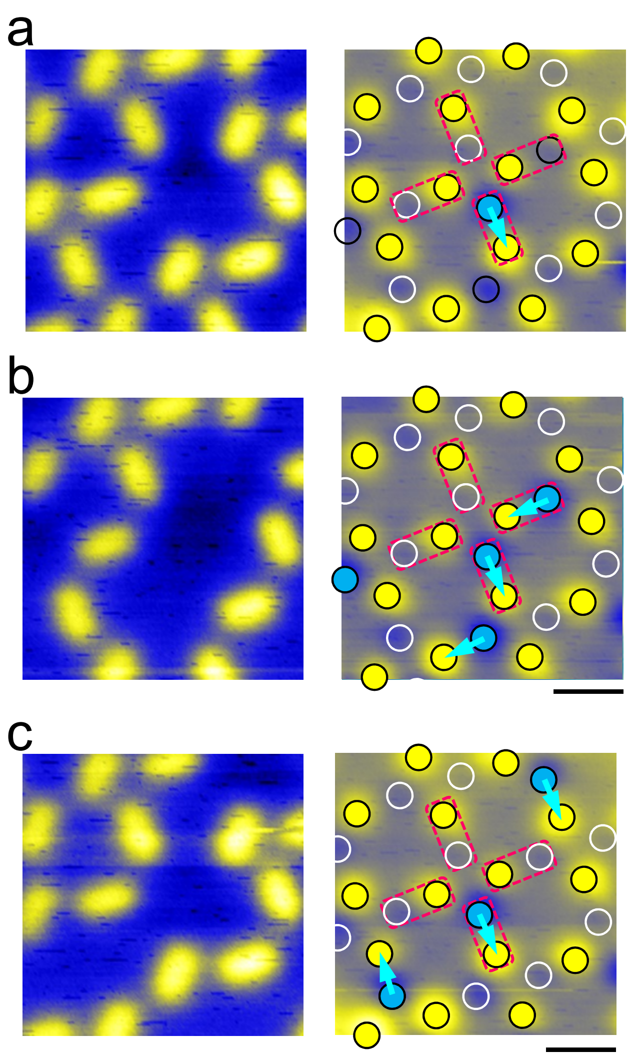

Supplementary Figure 7: Selectively erasing negative magnetic poles from the magnetic dipole pattern. In (a)-(c), the left hand side panels show the vortex distributions after field-cooling at magnetic fields smaller than the first matching field. After applying a Meissner current through the antidot lattice, a magnetic dipole pattern is overlapped with the vortex lattice. The right hand side panels demonstrate coexistence of vortex ice state with some remnant magnetic dipoles (indicated by the arrows). The circles indicate the positions where magnetic poles are expected to form after applying a supercurrent. The yellow/blue circles indicate the positive/negative magnetic poles. The open circles show the positions where negative magnetic poles are erased due to the overlap with the vortex lattice. The dashed rectangles correspond to one vertex of a square lattice.

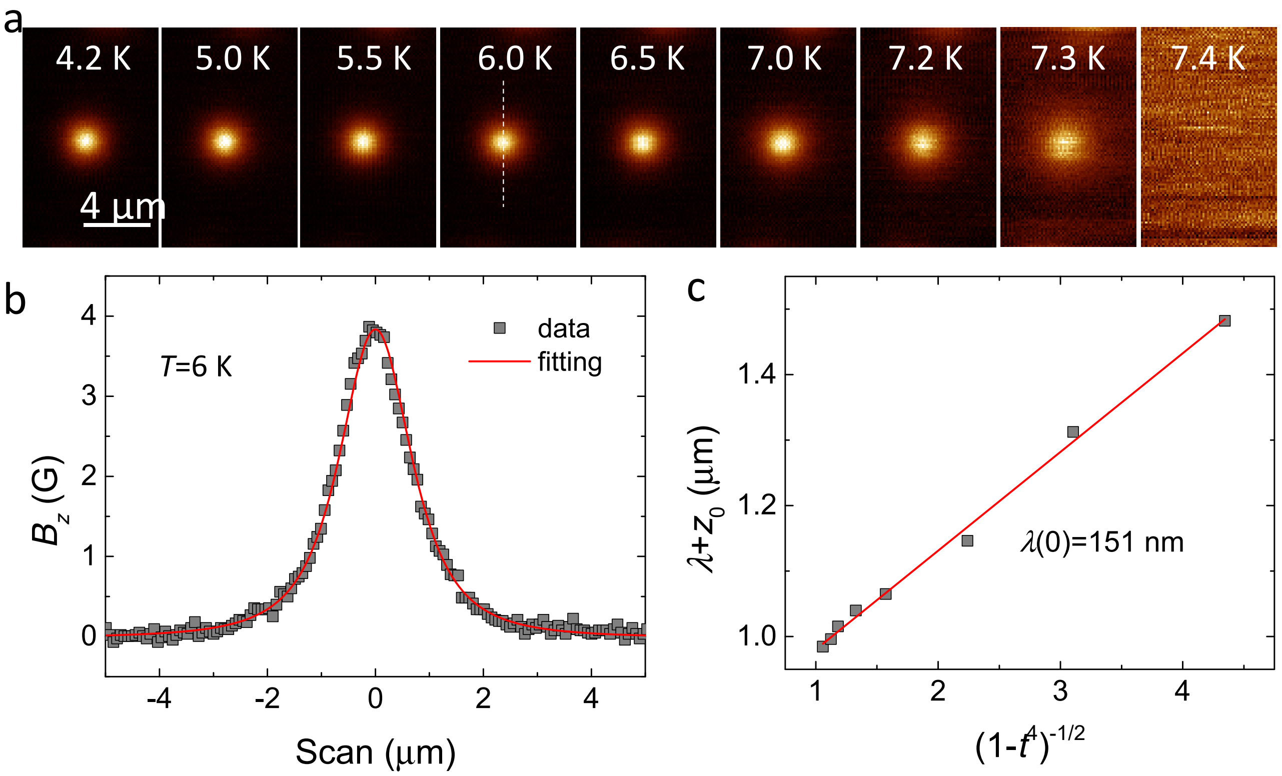

Supplementary Figure 8: Estimation of T_c and penetration detph. (a) Magnetic field distribution of a vortex at various temperatures. With increasing temperature, the vortex disappears between 7.3 K and 7.4 K. (b) Field profile along the dashed line for the vortex at T=6 K. Solid line in (b) is the monopole model fit. (c) $\lambda+z_0$ vs $(1-t^4)^{-1/2}$. Solid line in (c) is the linear fit to the data.

Supplementary Notes

Supplementary Note 1: Nature of the current source for the magnetic dipoles

The Meissner current is induced by applying an external magnetic field. For a superconducting stripe, the in-plane Meissner current can be expressed as[1, 2]:

$$J(x) = -2Hx(w^2 - x^2)^{-1/2}, \quad (1)$$

where w is the half width of the superconducting stripe, and x is the distance from its axis. The Meissner current flows along the edges of the sample.

For an unpinned vortex, the supercurrent (vortex current) circulate around its normal state core. At large distances from the vortex core, the current density on the surface of the superconductor follows the r^{-2} law which has a simple expression [3]:

$$J_s(r,0)=\Phi_0[\coth(d/\lambda)+\mathrm{csch}(d/\lambda)]/2\pi\mu_0\lambda r^2, \quad (2)$$

where r is the distance to the center of the vortex core, Φ_0 is the flux quantum, d is the sample thickness, λ is the penetration depth. For a vortex trapped by an elongated antidot with sizes larger than the vortex core, the supercurrent distribution is significantly modified: the current lines "expelled" from the antidot form two pronounced density maxima near the opposite ends of its long axis.

Since an antidot is much smaller than its distance from the nearest edge of the sample, the Meissner current density, given by Supplementary Eq. (1), remains nearly constant if variations of this distance are of the order of the antidot size. Flowing towards an elongated antidot, the Meissner current is redistributed to form two half-circular current flows. If the angle between the initial Meissner current and the long axis of the antidote is sufficiently large, the redistributed Meissner current density takes maximum values at the opposite ends of the antidot long axis – resembling, in a sense, the distribution of the trapped-vortex supercurrents. However, while at one end of the antidot, the Meissner current is added to the vortex supercurrent, enhancing the local magnetic field, at the other end of the antidot, the Meissner current and the vortex current tend to cancel each other so that the local field is suppressed.

We use the Meissner current instead of a transport current because both currents have qualitatively similar density distributions in the vicinity of the corresponding sample edges. At first sight, when using a transport current to generate magnetic patterns (one can even generate such magnetic patterns in the normal state, at room temperature), a more uniform distribution of magnetic dipoles may be expected. However, one should bear in mind that, in the Meissner state, due to the shielding of magnetic field, the transport supercurrent tends to have maximum density only at the edges of the superconductor [4]. As a result, we would observe a similar magnetic pattern as that induced by the Meissner current. However, we have noticed a recently published paper [5], where the authors studied the current distribution at high current densities. They

have shown that the transport current has a non-uniform distribution at low values, while a crossover to the uniform distribution occurs at critical current. For our present research, due to the imperfection of the sample edges, it is rather difficult to control the current at such high values. The main goal of this research is to introduce a new way to design geometric lattices of magnetic moments. The Meissner current seems quite suitable for this purpose.

Supplementary Note 2: Estimation of T_c and critical parameters

Enhanced T_c in superconducting films have already been reported in many materials. For our Pb film, we have determined T_c in two different ways. First, by measuring the local ac susceptibility, we observe T_c~7.15 K. Second, by directly imaging one vortex with slowly increasing temperature, we find that the vortex disappears between 7.3 K and 7.4 K (Supplementary Fig. 8a). Therefore, we determine the T_c of our sample as 7.35 K. Compared with transport and magnetization measurements on a bulk sample, our method gives a more precise estimate of T_c , due to the fact that, close to T_c, the vortex matter is already in the liquid regime and shielding of external magnetic field is rather weak. We notice that, T_c=7.3±0.05K for Pb has also been found in the carefully designed experiment of [6].

Pure bulk Pb is a well known type-I superconductor. In the present research, the sample is a superconducting film which was prepared with e-beam evaporation technique. The base pressure of the system is $2x10^{-8}$ Torr, and the pressure did not exceed 10^{-7} Torr during the evaporation. The evaporation rate is 1 $Ås^{-1}$. In order to ensure a uniform layer growth for the Pb, the substrate was cooled by using liquid nitrogen during the preparation.

Normally, the properties of superconducting films, prepared with the e-beam technique, strongly depend on the evaporation pressure, substrate temperature and so on. For example, the use of a cold (room temperature) substrate leads to formation of a dirty Nb film, while a heated substrate (780 ℃) results in the clean limit samples [7]. Our Pb sample is prepared at 77 K. As a result, a dirty limit sample is obtained with a relatively large penetration depth and small coherence length. Therefore, the parameters of pure bulk Pb cannot be directly used to estimate the critical values of our thin film.

We have estimated the penetration depth and coherence length of our sample. The penetration depth is determined by using the monopole model [8] to fit the vortex field profile measured at various temperatures. Figure s8a shows the SHPM images taken at various temperatures indicated above each image. By fitting the magnetic field profiles along the dashed line 9 (the results for T = 6 K are shown in Supplementary Fig. 8b), we can get the value of $\lambda+z_0$ at different temperatures, where λ is the penetration depth and z_0 is the distance between the two-dimensional electron gas (TDEG) of our Hall cross and the sample surface (this distance is constant). According to the two-fluid model, the penetration depth follows a linear dependence on $(1-t^4)^{-1/2}$ with $t = T/T_c$. As shown in

Supplementary Fig. 8c, from the slope of the linear fitting line, we can get the penetration depth at zero temperature. From the fitting, we get λ(0)=151 nm.

To determine the coherence length, we measured the temperature dependence of the in-phase ac susceptibility at different magnetic fields. The temperature dependence of H_{c2} can be deduced from the χ'-T curve. The coherence length is calculated using the relation $H_{c2}(T)=\Phi/2\pi\xi(T)$. With the two-fluid model, we estimate ξ(0)=52 nm. Therefore, for the Ginzburg-Landau parameter we obtain the value $\kappa \approx 2.9$ that places our sample well in the type-II regime. Also, from the literature [9], we see that the critical thickness above which the Pb film transits from type-II to type-I superconductor is around *d*=300 nm. Moreover, we did not observe any giant vortices or normal domains in the reference sample. All the evidences above suggest that our sample is a type-II superconductor.

Supplementary References

[1] Huebener, R., Kampwirth, R., Clem, J. R. Meissner shielding currents and magnetic flux penetration in thin-film superconductors. *J. Low Temp. Phys.* **6**, 275–285 (1972).

[2] Zeldov, E., Larkin, A., Geshkenbein, V., Konczykowski, M., Majer, D., Khaykovich, B., Vinokur, V., Shtrikman, H. Geometrical Barriers in High-Temperature Superconductors. *Phys. Rev. Lett.* **73**, 1428 (1994).

[3] Wei, J.-C. and Yang, T.-J. Current distribution and Vortex-Vortex Interaction in a Superconducting Film of Finite Thickness. *Jpn. J. Appl. Phys.* **35**, 5696-5700 (1996)

[4] Bobyl, A. V., Shantsev, D. V., Galperin, Y. M., Johansen, T. H., Baziljevich, M., Karmanenko, S. F. Relaxation of transport current distribution in a YBaCuO strip studied by magneto-optical imaging. *Supercond. Sci. Technol.* **15**, 82–89 (2002)

[5] Talantsev, E. F., Pantoja, A. E., Crump, W. P. and Tallon, J. L. Current distribution across type II superconducting films: a new vortex-free critical state. *Sci. Rep.* **8**,1716 (2018).

[6] Westerdale, S. Superconducting Metals: Finding Critical Temperatures and Observing Phenomena. http://web.mit.edu/shawest/Public/jlab/Supercon/superpaper.pdf (2010)

[7] Peroz, C. and Villard, C. Flux flow properties of niobium thin films in clean and dirty superconducting limits. *Phys. Rev. B* **72**, 014515 (2005).

[8] Ge, J., Guterriez, J., Cuppens, J., Moshchalkov, V. V. Observation of single flux quantum vortices in the intermediate state of a type-I superconducting film. *Phys. Rev. B* **88**, 174503 (2013).

[9] Dolan, G. J. and Silcox, J. Critical Thicknesses in Superconducting Thin Films. *Phys. Rev. Lett.* **30**, 603-606 (1973).
